# Cardiac Complications Associated with Short-Term Mortality in Schizophrenia Patients Hospitalized for Pneumonia: A Nationwide Case-Control Study

**DOI:** 10.1371/journal.pone.0070142

**Published:** 2013-07-29

**Authors:** Ya-Tang Liao, Shu-Yu Yang, Hsing-Cheng Liu, Wei J. Chen, Chiao-Chicy Chen, Yen-Ni Hung, Chian-Jue Kuo

**Affiliations:** 1 Institute of Epidemiology and Preventive Medicine, College of Public Health, National Taiwan University, Taipei, Taiwan; 2 Taipei City Psychiatric Center, Taipei City Hospital, Taipei, Taiwan; 3 Graduate Institute of Clinical Pharmacy, College of Pharmacy, Kaohsiung Medical University, Kaohsiung, Taiwan; 4 Department of Psychiatry, School of Medicine, Taipei Medical University, Taipei, Taiwan; 5 Psychiatric Research Center, Taipei Medical University Hospital, Taipei, Taiwan; 6 Department of Psychiatry, Mackay Memorial Hospital, Taipei, Taiwan; 7 Department of Psychiatry, Mackay Medical College, Taipei, Taiwan; 8 Department of Nursing, School of Nursing, National Yang-Ming University, Taipei, Taiwan; 9 School of Gerontology Health management, College of Nursing, Taipei Medical University, Taipei, Taiwan; Beijing Institute of Microbiology and Epidemiology, China

## Abstract

**Background:**

Pneumonia is one of most prevalent infectious diseases worldwide and is associated with considerable mortality. In comparison to general population, schizophrenia patients hospitalized for pneumonia have poorer outcomes. We explored the risk factors of short-term mortality in this population because the information is lacking in the literature.

**Methods:**

In a nationwide schizophrenia cohort, derived from the National Health Insurance Research Database in Taiwan, that was hospitalized for pneumonia between 2000 and 2008 (n = 1,741), we identified 141 subjects who died during their hospitalizations or shortly after their discharges. Based on risk-set sampling in a 1∶4 ratio, 468 matched controls were selected from the study cohort (i.e., schizophrenia cohort with pneumonia). Physical illnesses were categorized as pre-existing and incident illnesses that developed after pneumonia respectively. Exposures to medications were categorized by type, duration, and defined daily dose. We used stepwise conditional logistic regression to explore the risk factors for short-term mortality.

**Results:**

Pre-existing arrhythmia was associated with short-term mortality (adjusted risk ratio [RR] = 4.99, p<0.01). Several variables during hospitalization were associated with increased mortality risk, including incident arrhythmia (RR = 7.44, p<0.01), incident heart failure (RR = 5.49, p = 0.0183) and the use of hypoglycemic drugs (RR = 2.32, p<0.01). Furthermore, individual antipsychotic drugs (such as clozapine) known to induce pneumonia were not significantly associated with the risk.

**Conclusions:**

Incident cardiac complications following pneumonia are associated with increased short-term mortality. These findings have broad implications for clinical intervention and future studies are needed to clarify the mechanisms of the risk factors.

## Introduction

Pneumonia is the leading cause of death due to infection [1], [2], and it accounts for more than 1 million hospitalizations annually in the United States at an estimated cost of 12.2 billion [3]. In Taiwan, about 160,000 are hospitalized annually for treatment of pneumonia [2]. Patients with schizophrenia who develop pneumonia are more likely to have poorer outcomes requiring more frequent ventilator treatment and carrying greater risk of acute respiratory failure than in the general population [4]. Furthermore, compared with the general population, patients with schizophrenia have excessive mortality attributable to natural causes [5]. Thus, as a basis for prevention, to recognize the risk factors associated with short-term mortality among schizophrenia patients with pneumonia is crucial for preventing premature deaths. We found limited paucity of data regarding these issues in the literature.

Short-term mortality among the schizophrenic patients with pneumonia can result from pre-existing comorbid physical illnesses [6], [7], [8], incident physical complications developing after the pneumonia [9], [10], drugs potentially associated with the development of pneumonia (such as antipsychotic drugs) [8], [11], and quality of care [4], [12].

Pre-existing physical comorbidities before the development of pneumonia could precipitate short-term death [13], and recent studies reported incident physical illnesses after pneumonia are associated with short-term mortality [9], [10], [14]. A recent meta-analysis [14] reported major cardiac complications occurred in a substantial proportion (17.7%) of patients with community-acquired pneumonia, including incident heart failure, acute coronary syndromes, and arrhythmias. Furthermore, incident cardiac complications are common in patients with community-acquired pneumonia and are associated with increased short-term mortality [9]. Additionally, a recent study [10] reported that serum glucose levels on admission to the hospital are predictive of short-term mortality in patients with community acquired pneumonia without pre-existing diabetes. Whether the pre-existing or incident physical illnesses after pneumonia predict short-term mortality among schizophrenic patients with pneumonia is still undetermined.

An emerging body of literature shows the association between antipsychotic drugs and the development of pneumonia in the elderly population [6], [7], in patients with schizophrenia [8], and in bipolar patients [11]. Several individual antipsychotics (such as clozapine, olanzapine, and quetiapine) are associated with incident pneumonia [8], [11]. Nonetheless, rare studies focused on the association between the antipsychotic drugs and short-term mortality.

We explored the risk factors associated with short-term mortality in a nested case-control study derived from a large, nationwide schizophrenia cohort hospitalized for pneumonia in Taiwan. We focused on the potential factors before and during hospitalization, including the drugs used, pre-existing and incident physical illnesses, and the characteristics of hospital care relative to the risk of short-term mortality, with the outlook of preventing premature deaths.

## Methods

### Data Source

The sampling cohort was obtained from the National Health Insurance (NHI) Research Database in Taiwan. The NHI program was launched on March 1, 1995 and contains registration files and original claims data for reimbursement. There are currently more than 23 million enrollees in the program representing 99.6% of Taiwan’s population in 2011 [11]. In 1996, the National Health Research Institute in Taiwan established the NHI Research Database.

### Ethics Statement

Individual identification is decoded for protection of confidentiality and all investigators signed an agreement guaranteeing patient confidentiality before using the database. This study was approved by the Institutional Review Board of the Committee on Human Subjects of Taipei City Hospital.

### Schizophrenia Cohort with Pneumonia as the Study Cohort

The schizophrenia cohort is described in detail in our previous study [8] and is briefly summarized here. The nationwide Psychiatric Inpatient Medical Claims database, a subset of the NHI research database, comprises a cohort of patients hospitalized for any psychiatric disorder (ICD-9-CM codes 290.xx to 319.xx) between 1996 and 2008 (N = 187,117), was used in this study.

We selected patients with at least one psychiatric admission between 2000 and 2008 but no psychiatric admissions between 1996 and 1999 (N = 125,225) from the Psychiatric Inpatient Medical Claims database. The inclusion criteria for the study subjects were at least one discharge diagnosis of schizophrenia (ICD-9-CM code 295.xx) and age at first admission of 18 to 65 years (N = 35,627). If a member of the schizophrenia cohort had a discharge diagnosis of pneumonia (ICD-9-CM codes 480.xx–486.xx and 507.xx) after their first admission, then they were included in this study (n = 1,741), and this group was defined as the study cohort. All of their medical records during 1999 and 2008 were retrieved [15]. A flow diagram of this study is provided in the supplementary material (Figure S1 in File S1).

### Short-Term Mortality Cases and their Controls

Among the study cohort, a total of 141 subjects died during their hospitalizations, or died shortly (within 30 days) after their discharges, as identified in the Registry for Beneficiaries database [16]; these subjects were defined as the cases with short-term mortality.

**Figure 1:**
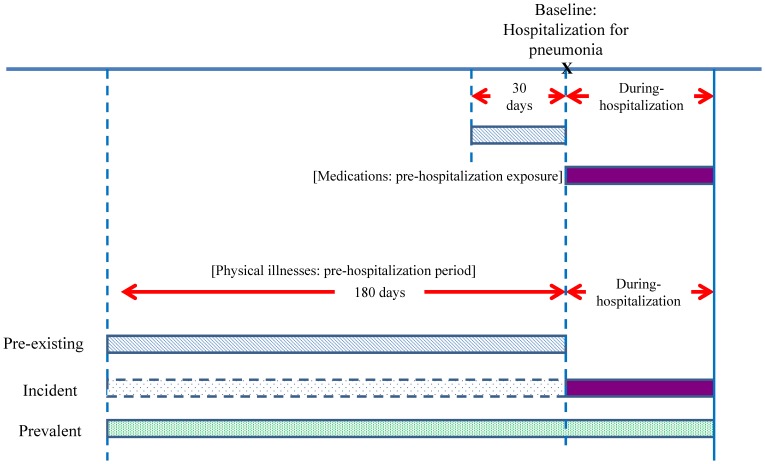
Overview for Study design.

The date of pneumonia hospitalization was defined as the baseline and the date of mortality was defined as the index date (Figure 1). Four controls or fewer (i.e., nonfatal pneumonia subjects) were randomly selected for each case from the cohort by risk set sampling, matching sex, age (within 5 years), and date of first psychiatric admission (within 1 year). Controls were assigned the same index date as their corresponding case. Finally, this study included a total of 128 case-control pairs (i.e., 128 cases and 468 controls) due to unavailability of controls for 13 cases.

### Drugs Exposure and Physical Illnesses

We obtained the medication histories of subjects for two periods (Figure 1): the period within 30 days before baseline (ie., the date of pneumonia requiring hospitalization) was defined as pre-hospitalization period for drug use. The medications included antipsychotic drugs and drugs administered for other medical conditions. Drugs were classified according to the Anatomical Therapeutic Chemical classification system [17]. The most common second-generation antipsychotics used by schizophrenic patients in Taiwan were selected, including clozapine, olanzapine, quetiapine, zotepine, risperidone, and amisulpride. First-generation antipsychotics included chlorpromazine, haloperidol, and sulpiride.

Physical illnesses were identified according to three definitions (Figure 1): pre-existing, incident, and prevalent illnesses. Pre-existing physical illnesses were an illness present within 180 days before the baseline date. Incident physical illness was an illness newly appearing after the baseline date. Those who had pre-existing or incident physical illnesses were defined as having prevalent physical illnesses.

Physical illnesses including cardiovascular disease (arrhythmia, heart failure, coronary heart failure, acute myocardial infarction, and hypertension), diabetes mellitus, cerebrovascular disease, chronic hepatic disease, cancer, asthma, upper respiratory tract infection, and delirium were studied for the association of risk with short-term mortality.

### Potential Risk Factors

Age and gender were controlled by the matching process of the study design. The Charlson comorbidity score [18], [19], which is the sum of the weighted scores of 31 comorbid conditions, at first psychiatric admission was used for adjustment of potential confounders in the analyses. The number of psychiatric hospitalizations within 180 days before the baseline was one of the covariates adjusted.

### Statistical Analysis

The crude incidence of short-term mortality was calculated as the number of incident cases divided by the contributed person-years for each individual in the cohort. Group comparisons between cases and controls were performed using univariate conditional logistic regression initially. Covariates with reasonable associations (p<0.1) were then entered into the final adjusted models. Multivariate regression with stepwise selection was used to estimate the adjusted models of association for short-term mortality.

Depending on the characteristics of drug exposure, physical illnesses, and potential risk factors at different time points during the course of pneumonia, three explanatory models were computed, including a pre-hospitalization model, during-hospitalization model 1, and during-hospitalization model 2. Incident physical illnesses were included in during-hospitalization model 1, while the prevalent illnesses were included in model 2.

Hospital characteristics, ownership, urbanization [20], and physician characteristics of age, gender, and specialty were included in during-hospitalization models 1 and 2, and are provided in the supplementary material (Table S1 in File S1). Multivariate models were calculated using SAS software, version 9.2 (SAS Institutes Inc., Cary, NC, USA). P values less than 0.01 were considered significant.

### Sensitivity Analysis

Recent studies [8], [11] showed that dose and duration of antipsychotic drugs are associated with increased risk of pneumonia. We conducted a sensitivity analysis for validating the risk of second-generation antipsychotic drugs on short-term mortality based on duration of use and cumulative defined daily dose within 30 days before hospitalization and during hospitalization. The results are provided in the supplementary materials (Table S2 in File S1, Table S3 in File S1).

## Results

### Incidence of Short-term Mortality

This study included 1741 schizophrenia patients with community-acquired pneumonia requiring hospitalization. Among the 141 cases with short-term mortality, 114 subjects died during hospitalization and 27 subjects died within 30 days after discharge. About 1 in 12 pneumonia patients (141/1741) died shortly after the development of pneumonia. The crude incidence of short-term mortality was calculated as the number of short-term mortality cases divided by the contributed person-years of all subjects (66,838 admission-days) in the cohort. The incidence of short-term mortality was 2.11 cases per 1,000 person-days (95% CI: 1.80–2.50, based on the Poisson distribution).

### Demographical and Clinical Characteristics of Cases and Controls

Analysis of demographic characteristics showed that there was no difference between cases and controls in gender, age, or Charlson comorbidity index (Table 1). The mean number of psychiatric hospital admissions among cases was significantly lower than among controls (0.5 vs. 0.7, p = 0.0013).

**Table 1:** Demographical and Clinical Characteristics of Cases with Short-Term Mortality and Controls Derived from a Nationwide Schizophrenia Cohort with Pneumonia Requiring Hospitalization.

Characteristic N (%)	Cases (n = 128)	Controls (n = 468)	Unadjusted risk ratio^a^	95% CI
At first psychiatric admission	*n (%)*	*n (%)*		
Men	72 (56.3)	275 (58.8)	–	–
Mean age (SD), years	48.5 (11.0)	48.4 (10.8)	1.00	0.89–1.12
Charlson comorbidity index				
0–1	99 (77.3)	344 (73.5)	Reference	
2	19 (14.8)	96 (20.5)	0.65	0.37–1.14
≥3	10 (7.8)	28 (6.0)	1.31	0.60–2.85
	*mean (SD)*	*mean (SD)*		
Number of psychiatric hospital admissionswithin 180 days before the baseline	0.5 (0.7)	0.7 (1.0)	0.61*	0.46–0.83
Duration between the baseline and the index date, days	13.3 (13.0)	11.9 (11.6)	–	–

a Estimated using univariate conditional logistic regression;

* p<0.01.

### Pre-existing, Incident, and Prevalent Physical Illness

Univariate analyses (Table 2) showed that rather than controls, cases had higher proportions of pre-existing arrhythmia, cancer, and chronic hepatic disease. Cases had higher proportions of incident physical illnesses only for arrhythmia (6.3% vs. 1.1%, p = 0.0014) and heart failure (4.7% vs. 1.1%, p = 0.0119) than did controls.

**Table 2:** Distribution of Physical Illnesses among Cases with Short-Term Mortality and Controls.

Characteristics, n (%)	Pre-existing illness^a^			Incident physical illness^b^			Prevalent physical illness^c^		
	Case (n = 128)	Controls (n = 468)	p^d^	Case (n = 128)	Controls (n = 468)	p^d^	Case (n = 128)	Controls (n = 468)	p^d^
Cardiovascular disease									
Arrhythmia	9 (7.0)	8 (1.7)*	<0.01	8 (6.3)	5 (1.1)*	<0.01	17 (13.3)	13 (2.8)**	<0.001
Heart failure	6 (4.7)	11 (2.4)	ns	6 (4.7)	5 (1.1)+	0.0119	12 (9.4)	16 (3.4)*	<0.01
Acute myocardial infarction	1 (0.8)	1 (0.2)	ns	2 (1.6)	1 (0.2)	ns	3 (2.3)	2 (0.4)	ns
Coronary heart diseases	8 (6.3)	32 (6.8)	ns	2 (1.6)	12 (2.6)	ns	10 (7.8)	44 (9.4)	ns
Hypertension	22 (17.2)	86 (18.4)	ns	1 (0.8)	12 (2.6)	ns	23 (18.0)	98 (20.9)	ns
Cancer	20 (15.6)	21 (4.5)**	<0.001	7 (5.5)	15 (3.2)	ns	27 (21.1)	36 (7.7)**	<0.001
Chronic hepatic disease	20 (15.6)	32 (6.8)*	<0.01	3 (2.3)	10 (2.1)	ns	23 (18.0)	42 (9.0)*	<0.01
Diabetes mellitus	22 (17.2)	63 (13.5)	ns	7 (5.5)	17 (3.6)	ns	29 (22.7)	79 (16.9)	ns
Cerebrovascular disease	7 (5.5)	34 (7.3)	ns	7 (5.5)	17 (3.6)	ns	14 (10.9)	51 (10.9)	ns
Asthma	4 (3.1)	19 (4.1)	ns	1 (0.8)	5 (1.1)	ns	5 (3.9)	24 (5.1)	ns
Delirium	1 (0.8)	3 (0.6)	ns	0	0	ns	1 (0.8)	3 (0.6)	ns

a Pre-existing physical illness: illness present within 180 days before hospitalization.

b Incident physical illness: the illness newly appeared after hospitalization.

c Prevalent physical illness: pre-existing and incident physical illnesses combined.

d Estimated using univariate conditional logistic regression.

ns: not significant (p>0.01),

* p<0.01,

** p<0.001,

+ marginal statistical significance.

Prevalent physical illnesses during hospitalization among cases revealed greater proportions of heart failure, arrhythmia, chronic hepatic disease, and cancer than among control cases.

### Medication Exposure

Table 3 summarizes the medication exposure in both the pre-hospitalization and during-hospitalization periods. Pre-hospitalization use of antipsychotic drugs did not differ between cases and controls, except for hypoglycemic drugs, which were used in a higher proportion among cases compared with controls with marginal statistical significance (p = 0.0254).

**Table 3:** Distribution of Antipsychotics and Other Medications Used in Cases with Short-Term Mortality and Controls.

	Pre-Hospitalization Period			During-Hospitalization Period		
Characteristics	Cases (n = 128)	Controls (n = 468)	p^a^	Cases (n = 128)	Controls (n = 468)	p^a^
	*n (%)*	*n (%)*		*n (%)*	*n (%)*	
**Antipsychotic drugs**						
Clozapine	13 (10.2)	69 (14.7)	ns	5 (3.9)	75 (16.0)**	<0.001
Olanzapine	9 (7.0)	31 (6.6)	ns	3 (2.3)	47 (10.0)*	<0.01
Quetiapine	17 (13.3)	42 (9.0)	ns	6 (4.7)	37 (7.9)	ns
Zotepine	5 (3.9)	31 (6.6)	ns	4 (3.1)	33 (7.1)	ns
Risperidone	23 (18.0)	105 (22.4)	ns	7 (5.5)	96 (20.5)**	<0.001
Amisulpride	1 (0.8)	16 (3.4)	ns	0 (0.0)	19 (4.1)	ns
Chlorpromazine	10 (7.8)	22 (4.7)	ns	4 (3.1)	28 (6.0)	ns
Haloperidol	22 (17.2)	78 (16.7)	ns	19 (14.8)	147 (31.4)**	<0.001
Sulpiride	14 (10.9)	73 (15.6)	ns	11 (8.6)	68 (14.5)	ns
**Other drugs**						
Cardiovascular drugs			–			–
Antihypertensive drugs	4 (3.1)	22 (4.7)	ns	3 (2.3)	23 (4.9)	ns
Beta blocking agents	22 (17.2)	119 (25.4)	ns	20 (15.6)	121 (25.9)	ns
Calcium channel blockers	16 (12.5)	55 (11.8)	ns	29 (22.7)	85 (18.2)	ns
Agents acting on the renin-angiotensinsystem	8 (6.3)	28 (6.0)	ns	6 (4.7)	40 (8.6)	ns
Lipid modifying agents	3 (2.3)	6 (1.3)	ns	1 (0.8)	7 (1.5)	ns
Hypoglycemic drugs	23 (18.0)	50 (10.7)	ns	44 (34.4)	80 (17.1)**	<0.001
Antithrombotic agents	12 (9.4)	37 (7.9)	ns	16 (12.5)	58 (12.4)	ns
Corticosteroids for systemic use	16 (12.5)	33 (7.1)	ns	62 (48.4)	126 (26.9)**	<0.001
Anti-Parkinson drugs	60 (46.9)	248 (53.0)	ns	27 (21.1)	205 (43.8)**	<0.001
Respiratory drugs	47 (36.7)	210 (44.9)	ns	98 (76.6)	374 (79.9)	ns
Benzodiazepines	59 (46.1)	249 (53.2)	ns	64 (50.0)	289 (61.8)*	<0.01

a Estimated using univariate conditional logistic regression.

ns: not significant (p>0.01),

* p<0.01,

** p<0.001.

Cases had lesser during-hospitalization use of multiple antipsychotic drugs (including clozapine, olanzapine, risperidone, and haloperidol) than controls. Relative to controls, cases used a greater proportion of hypoglycemic drugs and corticosteroids, while anti-Parkinson drugs and benzodiazepines were used in lower proportions.

### Multivariate Regression Model (Pre-hospitalization Model, During-hospitalization Models 1 and 2)

**Figure 2:**
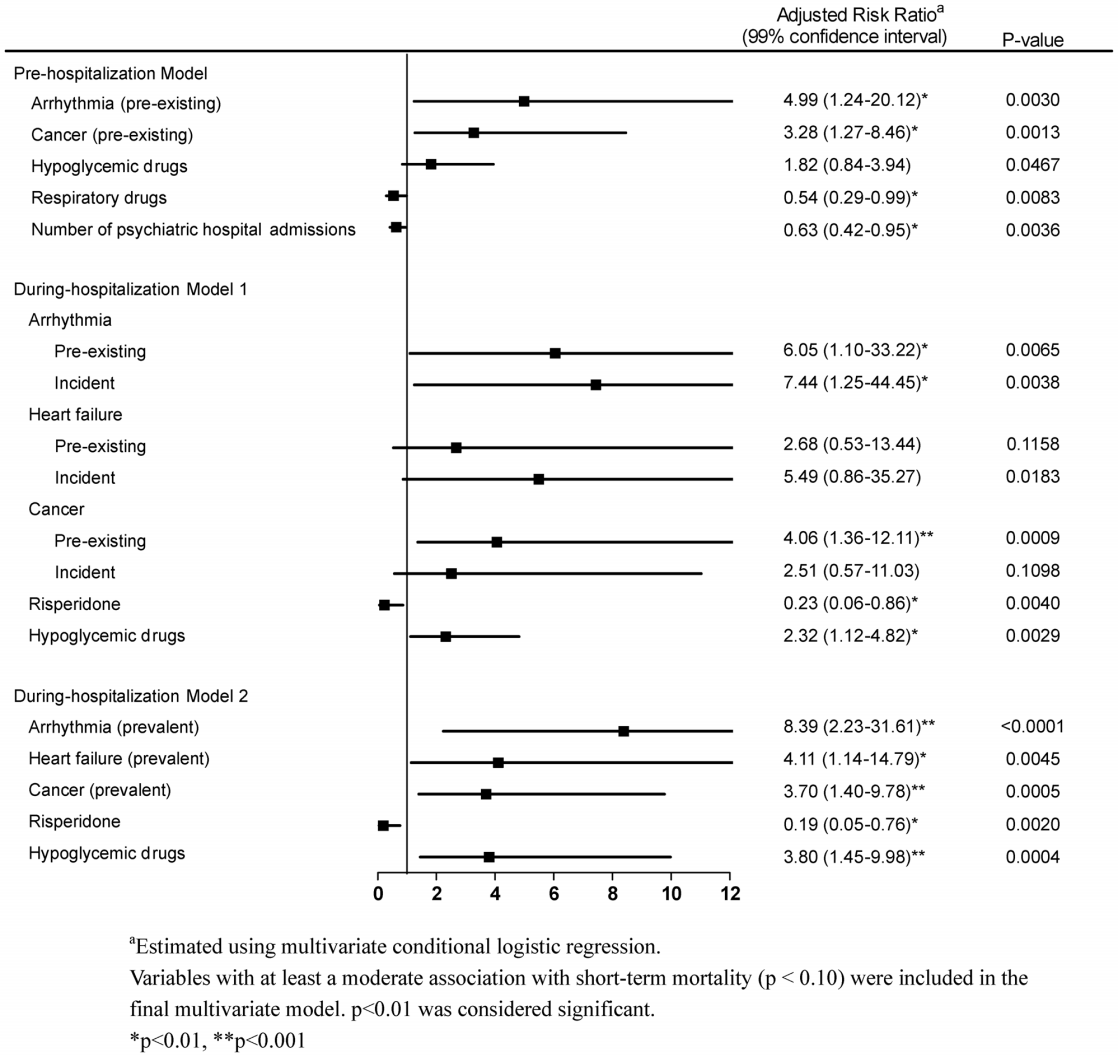
Multivariate Conditional Logistic Regression of Risk Factors for Short-Term Mortality among Schizophrenia Patients with Pneumonia Requiring Hospitalization.

In the pre-hospitalization model (Figure 2), several factors carried significantly increased risks of short-term mortality, specifically pre-existing arrhythmia and cancer, while the number of psychiatric hospital admissions and use of respiratory drugs indicated lower risk.

In during-hospitalization model 1, incident arrhythmia (RR = 7.44, p = 0.0038) greatly increased the risk of short-term mortality, while incident heart failure was associated with higher (RR = 5.49, p = 0.0183). Hypoglycemic drug use (RR = 2.32, p = 0.0029) was associated with a higher risk of short-term mortality, while risperidone use was associated with a lower risk.

In during-hospitalization model 2, prevalent physical illnesses including arrhythmia, heart failure, and cancer were associated with increased risk of short-term mortality. Hypoglycemic drug use showed an increased risk of short-term mortality, while the use of risperidone was associated with a lower risk.

### Sensitivity Analysis

Based on the duration and cumulative dose of pre-hospitalization drug use, the associations between second-generation antipsychotic drugs and risk of short-term mortality are summarized in the supplementary material (Table S2 in File S1). After adjustment, the results showed that the second-generation drugs had no association with increased risk of short-term mortality. Furthermore, the duration and dose of during-hospitalization second-generation antipsychotics use did not increase the risk of short-term mortality (Table S3 in File S1). These findings provide evidence strengthening the notion that second-generation antipsychotic drugs have no role in causing short-term mortality.

## Discussion

### Strengths

To our knowledge, this is the first nationwide case-control study to investigate the factors associated with short-term mortality in schizophrenia patients with pneumonia requiring hospitalization. This study revealed that a considerable portion of subjects died during their hospitalizations. We believe these patients deserve special clinical attention while hospitalized for pneumonia. By means a nested case-control study that is clear in temporal sequence, this study demonstrated that short-term mortality was associated with cardiac complications, such as arrhythmia and heart failure.

### Cardiac Complications

Despite the fact that pneumonia [9] is a leading cause of death, little consideration has been given to understanding the contributors to this mortality, especially among schizophrenia patients. Based on chronological order, the pre-hospitalization multivariate model provides clear evidence that pre-existing arrhythmia and cancer are risk factors for subsequent short-term mortality. A recent study [9] reported that pre-existing cardiovascular disease was associated with increased short-term mortality in patients with community-acquired pneumonia. We add to this the evidence of pre-existing arrhythmia before pneumonia, which predicted the short-term mortality.

Three prior studies [9], [21], [22] suggested incident cardiac complications following the pneumonia were associated with increased mortality in patients with community-acquired pneumonia, and one of them [9] adjusted for pneumonia severity showing a 1.6-fold risk for short-term mortality when patients had existing incident cardiac complications. Nonetheless, no prior studies showed the specific cardiac complications as predictors for short-term mortality, especially in non-elderly patients with schizophrenia. The present study adds robust evidence to identify incident arrhythmia and incident heart failure as the risk factors for short-term mortality among schizophrenia patients.

Pneumonia can induce several types of acute cardiac events through different mechanisms [21], including arrhythmia, congestive heart failure, acute myocardial infarction, and coronary heart disease. This provides possible explanation for the association between acute cardiac events and short-term mortality in pneumonia patients [21]. First, arrhythmia and congestive heart failure share similar pathogenesis, including increased myocardial demand for oxygen, lowered blood oxygen levels via ventilation-perfusion mismatch, and suppression of ventricular function by elevated levels of cytokines [21], [23]. Second, acute myocardial infarction is the most severe form of coronary heart disease. In this study, we could not prove the association between acute myocardial infarction and short-term mortality, which might be due to the low incidence of acute myocardial infarction among the schizophrenia patients with pneumonia. Coronary heart disease was distributed similarly between the cases and controls and, thus, is unlikely to be a predictor of short-term mortality.

The striking findings of this study demonstrated that specific cardiac complications, i.e., incident arrhythmia or incident heart failure, superimposed on pneumonia resulted in greater harm, especially short-term mortality, which was in line with the first proposed mechanism described above. Given this possible mechanism, incident arrhythmia or incident heart failure, which are pneumonia-related illnesses could be intermediate factors [14] and result in the short-term mortality. The possible mechanism deserves future work for clarification.

This study has few pneumonia cases developed incident physical illness after pneumonia and incident physical illnesses might exist before pneumonia happened with under diagnosis (especially in schizophrenia). Thus, future studies with larger sample size and better detection of incident physical illnesses before pneumonia are needed.

### Hypoglycemic Drugs

One of our major findings was that hypoglycemic drugs used during the hospitalization period were strongly associated with short-term mortality in the multivariate regression analyses. Use of hypoglycemic drugs reflects clinical hyperglycemia requiring treatment. This finding is in line with a recent study [10] that reported serum glucose levels on admission to the hospital can be used to predict death in patients with community-acquired pneumonia without pre-existing diabetes.

One of the limitations of our study was that we were unable to differentiate hyperglycemia during hospitalization into pre-existing hyperglycemia or incident hyperglycemia after the pneumonia. Our study did not show that patients with diabetes had a significantly increased risk of short-term mortality compared with patients without diabetes. Thus, we believe that hyperglycemia instead of diabetes mellitus is a sensitive marker for predicting short-term mortality. Given this possible mechanism [24], patients with hyperglycemia are prone to dehydration secondary to osmotic diuresis and exhibit a variety of perturbations of platelet function and endothelial function, as well as delayed chemotaxis, diminished granulocyte adherence, impaired phagocytosis, and reduced antimicrobial capacity [25], [26], [27], which could enhance the risk of mortality.

### Antipsychotic Use

A recent study [28] highlighted that the proportion of days covered with psychiatric medication in a year was positively associated with pneumonia but negatively associated with death. Prior studies [7], [8], [11] have further indicated that antipsychotic drugs contributed the development of pneumonia, especially clozapine and olanzapine [8], [11]. Nonetheless, based on multivariate regression, this study found that the use of antipsychotic drugs in both the pre-hospitalization and during-hospitalization periods were not associated with short-term mortality, except the use of risperidone during hospitalization period (Figure 2). This study has its potential limitation due to protopathic bias [29], i.e., the assignment of antipsychotics based on the patient’s clinical condition. One possible explanation was that during hospitalization, medication for chronic disease usually paused until acute symptoms were relieved. Thus, despite risperidone’s significantly negative association with short-term mortality, conservatively, we cannot conclude it has a protective effect against short-term mortality. In contrast, because antipsychotic drugs are associated with a risk of pneumonia, whether the use of antipsychotics during hospitalization should be continued or discontinued, or continued at a low dose to reduce the risk of pneumonia deserves further research. Furthermore, the causes and types of pneumonia were not specified in this study. The direct link between use of antipsychotics and short-term mortality is still awaiting further clarification.

### Hospital Care

Although prior studies [4], [12], [30] reported poor care quality was associated with mortality during hospitalization for pneumonia, the characteristics of hospital care were not associated with short-term mortality among schizophrenia patients hospitalized for pneumonia in our study (Table S1 in File S1).

### Limitations

This study is limited in several ways. First, our study did not cover data regarding the pneumonia severity index [9] as well as causes and types of pneumonia, which could, along with the protopathic bias, influence the prescribing behavior of doctors. Nonetheless, the estimation of the association between incident physical illnesses and risk of short-term mortality was less biased due to the nature of data being unrelated to protopathic bias.

Second, body weight and laboratory data were unavailable in the claims database. Potential unmeasured confounders or mediators included obesity, neutropenia, and hyperglycemia. We used the hypoglycemic drugs as the surrogate to reflect the clinical condition of hyperglycemia.

Third, the analyses did not include psychiatric severity data obtained from the claims data. Nonetheless, the number of prior psychiatric hospitalizations (a surrogate for severity) was removed in the multivariate analysis, providing evidence that short-term mortality could be independent of psychiatric severity.

Furthermore, a recent study [28] demonstrated that incidence and all-cause mortality of pneumonia in patients with schizophrenia were age-dependent and sex-specific. In this study, we used sex and age as matching variables to rule out the potential effects of these two variables and crystallized risk factors for short-term mortality of schizophrenia patients with pneumonia. However, sample selection bias or type I error due to small sample size could not be ruled out and limit the generalization and practicability.

### Implications

In summary, our study demonstrates that pre-existing arrhythmia predicts subsequent short-term mortality. Furthermore, incident arrhythmia following pneumonia has an independent impact on short-term mortality, so does incident heart failure. Additionally, hyperglycemia during hospitalization is another risk factor. Antipsychotic drugs with known induction of pneumonia were not associated with risk of mortality. Clinicians must recognize the burden of incident arrhythmia and heart failure among schizophrenia patients with pneumonia and keep clinically vigilant for their timely recognition.

## Supporting Information

File S1 (DOC)

## References

[1] Hoyert DL, Arias E, Smith BL (2001) Deaths: Final Data for 1999. Natl Vital Statistics Report 49: 1–113. 11591077

[2] Lin HC, Xirasagar S, Chen CH, Hwang YT (2008) Physician’s case volume of intensive care unit pneumonia admissions and in-hospital mortality. Am J Respir Crit Care Med 177: 989–994. 18263804 10.1164/rccm.200706-813OC

[3] Colice GL, Morley MA, Asche C, Birnbaum HG (2004) Treatment costs of community-acquired pneumonia in an employed population. Chest 125: 2140–2145. 15189934 10.1378/chest.125.6.2140

[4] Chen YH, Lin HC (2011) Poor clinical outcomes among pneumonia patients with schizophrenia. Schizophr Bull 37: 1088–1094. 20339152 10.1093/schbul/sbq019 PMC3160214

[5] Chen YH, Lee HC, Lin HC (2010) Mortality among psychiatric patients in Taiwan–results from a universal National Health Insurance programme. Psychiatry Res 178: 160–165. 20452059 10.1016/j.psychres.2008.07.023

[6] Knol W, van Marum RJ, Jansen PA, Souverein PC, Schobben AF (2008) Antipsychotic drug use and risk of pneumonia in elderly people. J Am Geriatr Soc 56: 661–666. 18266664 10.1111/j.1532-5415.2007.01625.x

[7] Trifiro G, Gambassi G, Sen EF, Caputi AP, Bagnardi V (2010) Association of community-acquired pneumonia with antipsychotic drug use in elderly patients: a nested case-control study. Ann Intern Med 152: 418–425, W139–440. 10.7326/0003-4819-152-7-201004060-00006 20368647

[8] Kuo CJ, Yang SY, Liao YT, Chen WJ, Lee WC (2013) Second-generation antipsychotic medications and risk of pneumonia in schizophrenia. Schizophr Bull 39: 648–657. 22282455 10.1093/schbul/sbr202 PMC3627761

[9] Corrales-Medina VF, Musher DM, Wells GA, Chirinos JA, Chen L (2012) Cardiac complications in patients with community-acquired pneumonia: incidence, timing, risk factors, and association with short-term mortality. Circulation 125: 773–781. 22219349 10.1161/CIRCULATIONAHA.111.040766

[10] Lepper PM, Ott S, Nuesch E, von Eynatten M, Schumann C (2012) Serum glucose levels for predicting death in patients admitted to hospital for community acquired pneumonia: prospective cohort study. BMJ 344: e3397. 22645184 10.1136/bmj.e3397 PMC3362658

[11] Yang SY, Liao YT, Liu HC, Chen WJ, Chen CC (2013) Antipsychotic drugs, mood stabilizers, and risk of pneumonia in bipolar disorder: a nationwide case-control study. J Clin Psychiatry 74: e79–86. 23419234 10.4088/JCP.12m07938

[12] Welte T, Torres A, Nathwani D (2012) Clinical and economic burden of community-acquired pneumonia among adults in Europe. Thorax 67: 71–79. 20729232 10.1136/thx.2009.129502

[13] Yende S, van der Poll T, Lee M, Huang DT, Newman AB (2010) The influence of pre-existing diabetes mellitus on the host immune response and outcome of pneumonia: analysis of two multicentre cohort studies. Thorax 65: 870–877. 20861291 10.1136/thx.2010.136317 PMC3306240

[14] Corrales-Medina VF, Suh KN, Rose G, Chirinos JA, Doucette S (2011) Cardiac complications in patients with community-acquired pneumonia: a systematic review and meta-analysis of observational studies. PLoS Med 8: e1001048. 21738449 10.1371/journal.pmed.1001048 PMC3125176

[15] Gau SS, Chung CH, Gau CS (2008) A pharmacoeconomic analysis of atypical antipsychotics and haloperidol in first-episode schizophrenic patients in Taiwan. J Clin Psychopharmacol 28: 271–278. 18480683 10.1097/JCP.0b013e3181723713

[16] Tung YC, Chang GM, Chen YH (2009) Associations of physician volume and weekend admissions with ischemic stroke outcome in Taiwan: a nationwide population-based study. Med Care 47: 1018–1025. 19648828 10.1097/MLR.0b013e3181a81144

[17] WHO-Collaborating-Centre-for-Drug-Statistic-Methodology. (2009) ATC index with DDDs. Oslo: WHO.

[18] Quan H, Sundararajan V, Halfon P, Fong A, Burnand B (2005) Coding algorithms for defining comorbidities in ICD-9-CM and ICD-10 administrative data. Med Care 43: 1130–1139. 16224307 10.1097/01.mlr.0000182534.19832.83

[19] Quail JM, Lix LM, Osman BA, Teare GF (2011) Comparing comorbidity measures for predicting mortality and hospitalization in three population-based cohorts. BMC Health Serv Res 11: 146. 21663672 10.1186/1472-6963-11-146 PMC3127985

[20] Liu CY (2006) Incorporating Development Stratification of Taiwan Townships into Sampling Design of Large Scale Health Interview Survey. Journal of Health Management. Journal of Health Management 4: 1–22.

[21] Musher DM, Rueda AM, Kaka AS, Mapara SM (2007) The association between pneumococcal pneumonia and acute cardiac events. Clin Infect Dis 45: 158–165. 17578773 10.1086/518849

[22] Mandal P, Chalmers JD, Choudhury G, Akram AR, Hill AT (2011) Vascular complications are associated with poor outcome in community-acquired pneumonia. QJM 104: 489–495. 21217116 10.1093/qjmed/hcq247

[23] Kumar A, Thota V, Dee L, Olson J, Uretz E Tumor necrosis factor alpha and interleukin 1beta are responsible for in vitro myocardial cell depression induced by human septic shock serum. J Exp Med 183: 949–958. 8642298 10.1084/jem.183.3.949 PMC2192364

[24] McAlister FA, Majumdar SR, Blitz S, Rowe BH, Romney J (2005) The relation between hyperglycemia and outcomes in 2,471 patients admitted to the hospital with community-acquired pneumonia. Diabetes Care 28: 810–815. 15793178 10.2337/diacare.28.4.810

[25] Oliver MF, Opie LH (1994) Effects of glucose and fatty acids on myocardial ischaemia and arrhythmias. Lancet 343: 155–158. 7904009 10.1016/s0140-6736(94)90939-3

[26] Williams SB, Goldfine AB, Timimi FK, Ting HH, Roddy MA (1998) Acute hyperglycemia attenuates endothelium-dependent vasodilation in humans in vivo. Circulation 97: 1695–1701. 9591763 10.1161/01.cir.97.17.1695

[27] McMahon MM, Bistrian BR (1995) Host defenses and susceptibility to infection in patients with diabetes mellitus. Infect Dis Clin North Am 9: 1–9. 7769211

[28] Chou FH, Tsai KY, Chou YM (2013) The incidence and all-cause mortality of pneumonia in patients with schizophrenia: a nine-year follow-up study. J Psychiatr Res 47: 460–466. 23317876 10.1016/j.jpsychires.2012.12.007

[29] Feinstein AR (1985) Clinical Epidemiology: The Architecture of Clinical Research. Philadelphia, PA: WB Saunders.

[30] Song JH, Thamlikitkul V, Hsueh PR (2011) Clinical and economic burden of community-acquired pneumonia amongst adults in the Asia-Pacific region. Int J Antimicrob Agents 38: 108–117. 21683553 10.1016/j.ijantimicag.2011.02.017

