# Supplementary material for: Cardiac Complications Associated with Short-Term Mortality in Schizophrenia Patients Hospitalized for Pneumonia: A Nationwide Case-Control Study
Source: PLoS One. 2013 Jul 29;8(7):e70142. doi: 10.1371/journal.pone.0070142 (PMC3726532; doi:10.1371/journal.pone.0070142)
Supplement: File S1 — (DOC) [file pone.0070142.s001.doc]

**Supporting Information Legends**

Figure S1. Study flow diagram

Patients with at least one discharge diagnosis of schizophrenia, from 2000–2008 (N=38,120)

Patients aged 18–65 years (N=35,627)

The study cohort: patients with incident hospitalized pneumonia after their first admissions (n=1,741)

Nationwide Psychiatric Inpatient Medical Claims (PMID) in Taiwan (ICD-9 code of 290.xx–319.xx) (1996–2008) (N=187,117)

Cases (n=141) who died during hospitalization or died shortly (within 30 days) after discharge

Excluding at least one diagnosis of mood disorder (ICD-9 code: 296.**) (N=87,105)

Patients first discharged with mental disorder diagnosis (ICD-9 code of 290.xx–319.xx), from 2000–2008 (N=125,225) (no psychiatric admissions from 1996–1999)

Valid case-control pairs:

128 cases and 468 controls (unavailability of controls for 13 cases)

Four controls for each case, matched for sex, age (within 5 years), and date of first psychiatric admission (within 1 year)

Table S1. Hospital Characteristics of Cases with Short-term Mortality and Their Controls

| Characteristic, n (%) | Cases  (n=128) | Controls  (n=468) | Unadjusted risk ratio^a^ | 95% CI | p |
| --- | --- | --- | --- | --- | --- |
|  | *n (%)* | *n (%)* |  |  |  |
| Hospital type |  |  |  |  |  |
| Medical center | 41 (32.0) | 110 (23.5) | Reference |  |  |
| Regional hospital | 53 (41.4) | 223 (47.7) | 0.66 | 0.41–1.05 | 0.0765 |
| District hospital | 34 (26.6) | 135 (28.9) | 0.71 | 0.42–1.21 | 0.2102 |
| Urbanization, n (%) |  |  |  |  |  |
| Highly urbanized Cities/townships | 37 (29.4) | 141 (30.5) | Reference |  |  |
| Medium urbanized Cities/townships | 61 (48.4) | 184 (39.7) | 1.33 | 0.82–2.14 | 0.2506 |
| Emerging cities/townships | 8 (6.4) | 42 (9.1) | 0.77 | 0.33–1.79 | 0.5503 |
| Ordinarily urbanized Cities/townships | 16 (12.7) | 53 (11.5) | 1.24 | 0.63–2.47 | 0.5363 |
| Agricultural townships | 4 (3.2) | 43 (9.3) | 0.38 | 0.13–1.13 | 0.0816 |
|  |  |  |  |  |  |
| Physician characteristics |  |  |  |  |  |
| Gender |  |  |  |  |  |
| Male | 122 (95.3) | 427 (91.2) | Reference |  |  |
| Female | 6 (4.7) | 41 (8.8) | 0.52 | 0.21–1.30 | 0.1605 |
| Age |  |  |  |  |  |
| <40 | 53 (41.4) | 188 (40.2) | Reference |  |  |
| 40–49 | 42 (32.8) | 184 (39.3) | 0.76 | 0.48–1.22 | 0.2590 |
| >49 | 33 (25.8) | 96 (20.5) | 1.23 | 0.73–2.05 | 0.4360 |
| Physician specialty |  |  |  |  |  |
| Pulmonary or critical care medicine | 5 (3.9) | 23 (4.9) | Reference |  |  |
| Other | 123 (96.1) | 445 (95.1) | 1.18 | 0.43–3.25 | 0.7529 |
|  |  |  |  |  |  |

^a^Estimated using univariate conditional logistic regression

Table S2. Duration of Therapy and Dosage of Individual Antipsychotic Drugs Used Within 30 days Before Baseline of Cases with Short-term Mortality and Their Controls

|  |  |  | Cases  (n=128) |  |  | Controls  (n=468) |  |  | Adjusted Model^a^ |  |
| --- | --- | --- | --- | --- | --- | --- | --- | --- | --- | --- |
| Characteristics |  | n  (use) | Mean (SD) of all cases |  | n  (use) | Mean (SD) of all controls |  | Adjusted  risk ratio | 95% CI | p |
| Duration of use within 30 days before baseline, days |  |  |  |  |  |  |  |  |  |  |
| Clozapine |  | 11 | 0.7 (4.1) |  | 29 | 2.6 (7.5) |  | 0.95 | 0.90–1.01 | 0.0759 |
| Olanzapine |  | 6 | 0.6 (3.4) |  | 11 | 1.0 (4.5) |  | 0.95 | 0.88–1.02 | 0.1659 |
| Quetiapine |  | 12 | 1.3 (5.2) |  | 25 | 1.1 (5.0) |  | 1.00 | 0.96–1.05 | 0.9442 |
| Zotepine |  | 2 | 0.6 (3.8) |  | 13 | 0.9 (4.2) |  | 1.01 | 0.95–1.07 | 0.8798 |
| Risperidone |  | 17 | 1.5 (5.6) |  | 49 | 3.5 (8.7) |  | 0.95 | 0.91–0.99 | 0.0230 |
| Amisulpride |  | 1 | 0.0 (0.0) |  | 4 | 0.7 (4.1) |  | 0.20 | – | 0.9923 |
|  |  |  |  |  |  |  |  |  |  |  |
| Cumulative defined daily dose within 30 days before baseline |  |  |  |  |  |  |  |  |  |  |
| Clozapine |  | 11 | 0.3 (1.8) |  | 29 | 1.7 (6.1) |  | 0.91 | 0.83–1.01 | 0.0831 |
| Olanzapine |  | 6 | 0.1 (0.7) |  | 11 | 0.9 (4.7) |  | 0.69 | 0.51–0.93 | 0.0153 |
| Quetiapine |  | 12 | 1.0 (4.8) |  | 25 | 0.8 (5.1) |  | 1.01 | 0.96–1.05 | 0.8251 |
| Zotepine |  | 2 | 0.6 (4.2) |  | 13 | 0.6 (3.4) |  | 1.02 | 0.96–1.08 | 0.5381 |
| Risperidone |  | 17 | 1.0 (4.4) |  | 49 | 2.2 (6.5) |  | 0.95 | 0.90–1.00 | 0.0573 |
| Amisulpride |  | 1 | 0.0 (0.0) |  | 4 | 1.1 (7.4) |  | 0.00 | – | 0.9888 |
|  |  |  |  |  |  |  |  |  |  |  |

^a^Adjusted for Charlson comorbidity index at the first admission, number of psychiatric hospital admissions within 180 days before baseline, and the following variables before baseline of 30 days, including pre-existing physical illnesses and pre-hospitalization use of concomitant medications.

Table S3. Duration of Therapy and Dosage of Individual Antipsychotic Drugs Used During the Hospitalization of Cases with Short-term Mortality and Their Controls

|  |  |  | Cases  (n=128) |  |  | Controls  (n=468) |  |  | Adjusted Model^a^ |  |
| --- | --- | --- | --- | --- | --- | --- | --- | --- | --- | --- |
| Characteristic, n (%) |  | n  (use) | Mean (SD) of all cases |  | n  (use) | Mean (SD) of all controls |  | Adjusted  risk ratio | 95% CI | p |
| Duration of use between baseline and the index date, days |  |  |  |  |  |  |  |  |  |  |
| Clozapine |  | 5 | 0.4 (2.8) |  | 75 | 2.1 (7.7) |  | 0.94 | 0.87–1.01 | 0.0931 |
| Olanzapine |  | 3 | 0.3 (2.2) |  | 47 | 1.3 (5.4) |  | 0.88 | 0.79–0.99 | 0.0257 |
| Quetiapine |  | 6 | 0.7 (3.8) |  | 37 | 1.1 (6.0) |  | 0.99 | 0.94–1.04 | 0.7048 |
| Zotepine |  | 4 | 0.5 (3.3) |  | 33 | 0.8 (3.9) |  | 1.03 | 0.97–1.10 | 0.3118 |
| Risperidone |  | 7 | 1.0 (6.5) |  | 96 | 2.6 (7.4) |  | 0.95 | 0.90–1.00 | 0.0440 |
| Amisulpride |  | 0 | 0.0 (0.0) |  | 19 | 0.4 (2.5) |  | 0.00 | – | 0.9902 |
|  |  |  |  |  |  |  |  |  |  |  |
| Cumulative defined daily dose from baseline to index date |  |  |  |  |  |  |  |  |  |  |
| Clozapine |  | 5 | 0.1 (0.5) |  | 75 | 0.8 (4.0) |  | 0.74 | 0.52–1.05 | 0.0902 |
| Olanzapine |  | 3 | 0.0 (0.4) |  | 47 | 1.0 (5.3) |  | 0.64 | 0.40–1.03 | 0.0668 |
| Quetiapine |  | 6 | 0.0 (0.3) |  | 37 | 0.5 (3.3) |  | 0.75 | 0.48–1.17 | 0.2099 |
| Zotepine |  | 4 | 0.2 (1.7) |  | 33 | 0.4 (2.6) |  | 1.02 | 0.92–1.13 | 0.7654 |
| Risperidone |  | 7 | 0.6 (5.5) |  | 96 | 1.4 (4.7) |  | 0.93 | 0.87–1.00 | 0.0602 |
| Amisulpride |  | 0 | 0.0 (0.0) |  | 19 | 0.3 (2.2) |  | 0.00 | – | 0.9918 |
|  |  |  |  |  |  |  |  |  |  |  |

^a^Adjusted for Charlson comorbidity index at the first admission, number of psychiatric hospital admissions within 180 days before baseline, and the following variables between baseline and the index date, including pre-existing physical illnesses and pre-hospitalization use of concomitant medications.
